# Factors influencing students’ satisfaction with continuous use of learning management systems during the COVID-19 pandemic: An empirical study

**DOI:** 10.1007/s10639-021-10492-5

**Published:** 2021-04-06

**Authors:** Latifa Alzahrani, Kavita Panwar Seth

**Affiliations:** 1 Department of Management Information Systems, College of Business Administration, Taif University, P.O. Box 11099, Taif, 21944 Saudi Arabia; 2 College of Business, Arts and Social Sciences, Brunel Business School, Brunel University London, London, UK

**Keywords:** Learning management systems, COVID-19, Student satisfaction, Continuous use, Higher education

## Abstract

COVID-19 has impacted educational processes in most countries: some educational institutions have closed, while others, particularly in higher education, have converted to online learning systems, due to the advantages offered by information technologies. This study analyzes the critical factors influencing students’ satisfaction with their continuing use of online learning management systems in higher education during the COVID-19 pandemic. Through the integration of social cognitive theory, expectation confirmation theory, and DeLone and McLean’s IS success model, a survey was conducted of 181 UK students who engaged with learning management systems. It was found that, during the pandemic, service quality did not influence students’ satisfaction, although both information quality and self-efficacy had significant impacts on satisfaction. In addition, the results revealed that neither self-efficacy nor satisfaction impacted personal outcome expectations, although prior experience and social influence did. The findings have practical implications for education developers, policymakers, and practitioners seeking to develop effective strategies for and improve the use of learning management systems during the pandemic.

## Introduction

The novel coronavirus has spread very rapidly all over the world, and has impacted almost everyone in some way. Higher education has also been affected by this pandemic. According to a 2020*Times Higher Education* survey, universities’ finances might suffer, and university leaders must answer key questions, such as whether they should prepare universities for increased online education and accept that blended learning will be the new normal. Students are questioning whether they might be able to return to campus anytime soon, potentially inducing various physical, mental, and emotional issues (Li et al., 2020; Yang et al., 2020).

Online learning or (E-learning) is defined as "the use of electronic media for a variety of learning purposes that range from add-on functions in conventional classrooms to full substitution for the face-to-face meetings by online encounters” (Guri-Rosenblit, 2005). According to Zhang et al. (2004), e-learning is defined as “technology-based learning in which learning materials are delivered electronically to remote learners via a computer network” (p. 76). Learning management systems (LMSs), meanwhile, are considered as one of the most widely used applications in higher education institutions to support course activities in the digital environment (Fındık-Coşkunçay et al., 2018).

Many factors can impact online learning and its success or failure, as students feel engaged and rewarded while attending online courses (Moawad, 2020). Rovai & Downey (2010) demonstrate that teachers must be well-trained, and the resources should be useful. Online teaching might be unsuccessful if there is a lack of trust in fully online courses or a “wide gap between management and the Deanship of E-learning and Distance Education” (Aljaber, 2018). Aljeraiwi & Sawaftah (2018) observed universities using Google Class, Zoom, and Blackboard (an online teaching platform that allows chat, resource sharing, and video calls between teachers and students) to create effective LMSs, and found issues such as technological difficulties caused barriers for students and faculty. Inappropriate infrastructure and lack of technical support were considered the main concerns while using Blackboard and online learning. However, the key benefit of online learning is that students can access information anytime and anywhere.

Most recent studies in the field of e-learning have focused on the technical aspects of information technology and given limited consideration to social factors and those related to students (Fındık-Coşkunçay et al., 2018; Rovai & Downey, 2010). However, examining the critical factors influencing students’ satisfaction and their intention to use LMSs from multidimensional perspectives can provide a deep understanding that can help improve and develop online learning environments and the effective and successful use of LMSs. This research, therefore, attempts to provide a validated conceptual framework that integrates social cognitive theory (SCT), expectation confirmation theory (ECT), and DeLone and McLean’s IS success model (the D&M model) to investigate and analyze the impacts of various factors on UK students’ satisfaction and their intention to use LMSs during the COVID-19 pandemic.

## Literature review

### Online learning systems

ICT use is important for colleges that offer distance learning programs, and colleges have developed models to incorporate ICT into the learning cycle, thus bestowing students with knowledge and abilities that are adjusted to current and future society. According to recent studies by Alruwaie et al. (2020), Moawad (2020), and Fındık-Coşkunçay et al. (2018), colleges have attempted to execute or create e-learning frameworks that are tailored to their hierarchical structure and to utilize mixed learning in their courses. These e-learning frameworks offer noteworthy enhancements to the learning cycle and significantly lessen the negative impacts of conventional education techniques.

E-learning comprises an arranged educational experience at a higher education institution that provides study materials via an e-learning innovation and an internet browser, which can be absorbed by students in their own way. Moawad (2020) and Aljeraiwi & Sawaftah (2018) demonstrate that e-learning innovations repeat and adjust the traditional instructional segments: development, explicit content and philosophy, cooperation, backing, and evaluation. In addition, Moawad (2020), Aljaber (2018), and Ferdousi (2009) highlight that the stages of e-learning frameworks encourage the learning cycle and focus on its adaptability and the transformation of training techniques for individual learning styles. According to recent studies (e.g., Aljeraiwi & Sawaftah, 2018; Cantoni et al., 2004; Moawad, 2020), the noteworthy contrasts between traditional educational strategies and internet teaching techniques necessitate cautious development, checking, and control. Also, Dorobat (2014) reports that the term “blended” signifies the blend of a few instructional techniques: nonconcurrent and simultaneous, off-website and on-location, disconnected and on the web, individual and shared, and organized and non-organized. The most favorable application of the mixed learning idea is the adjustment of instructional strategies to the individual’s learning style.

### Theoretical background

A systematic literature review was conducted to examine the critical factors influencing the effective use of LMSs in higher education. Most recent studies have paid significant attention to the technical aspects of LMSs, with limited focus on other aspects, such as social factors and students’ expectations and experiences (Table 1). Additionally, the technology acceptance model (TAM) has been the most widely employed to study the effective use of e-learning.

**Table 1:** Summary of selected recent studies on LMSs

No	Title and Author Name(s)	Research Aim	Research Model	Factors Influencing Student Intention	Results/Outcome
1	Ohliati & Abbas (2019)	This study aimed to determine the factors influencing student satisfaction with a learning management system at a private university that offers online learning	D&M Model TAM	• System quality • Service quality • Information quality • Perceived usefulness • Perceived ease of use • Communication quality	Information quality, service quality, and perceived ease of use had a significant effect on student satisfaction. Service quality was the most dominant factor affecting their satisfaction with the learning management system
2	Fındık-Coşkunçay et al. (2018)	This study identified the factors affecting higher education students’ behavioral intention toward learning management systems. A research model was proposed based on the belief factors of the technology acceptance model	TAM D&M Model Self-determination Theory TRA	• Perceived usefulness • Perceived ease of use • Self-efficacy, enjoyment • Subjective norms, satisfaction • Interactivity and control	The predictors of behavioral intention were defined through the validated structural model as perceived usefulness, perceived ease of use, enjoyment, subjective norms, satisfaction, and interactivity and control

#### Technology acceptance model

The TAM is an information systems theory that models how users accept and use a new technology. The model highlights that clients are impacted by two specific elements when they choose how and when they are going to utilize an innovation:

- “Perceived usefulness” or the degree to which a client accepts that, by utilizing a specific framework, they will acquire expanded proficiency.
- “Perceived ease of use” or the degree to which a client accepts that they will need significantly fewer attempts to satisfy their present undertaking when utilizing this framework.

#### DeLone and McLean’s IS success model

**Fig. 1:**
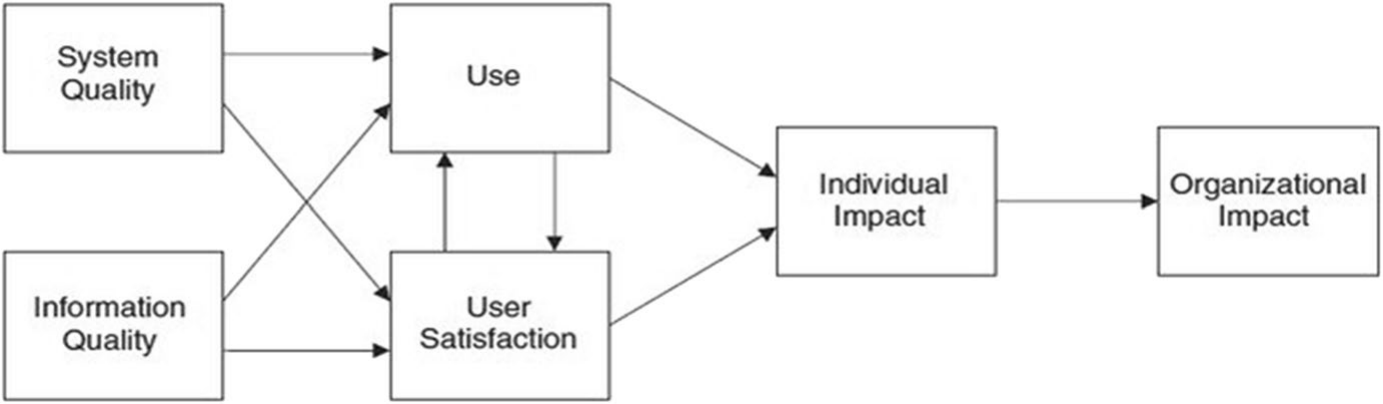
The D&M model (DeLone & McLean, 1992)

The D&M model was initially created by DeLone & McLean (1992) and later adjusted and evaluated by various analysts (e.g., DeLone & McLean, 2003; Holsapple & Lee-Post, 2006; Lin, 2008; Wang et al., 2007). It has been one of the most productive models for estimating the effectiveness of e-learning frameworks and has been used in more than 300 studies (DeLone & McLean, 2003). It incorporates six segments: the nature of the framework, the nature of the data, the utilization of the framework, client fulfillment, individual effect, and hierarchical effects. The relations between them are illustrated in Fig. 1.

Prior to 2003, relationships between the model’s segments were the subject of a few logical examinations (DeLone & McLean, 2003), 16 of which were recognized by DeLone and McLean (Holsapple & Lee-Post, 2006), and the model was subsequently updated in 2003 by its creators. The model incorporates six measurements (DeLone & McLean, 2003; Wang et al., 2007): framework quality, data quality, administration quality, aim to utilize or utilization of the framework, client fulfillment, and the advantages of utilizing the framework. However, use of the D&M model for estimating the success of e-learning frameworks has been censured variously, such as it does not consider cultural viewpoints, the instructor’s point of view, the connection between the model parts (Wang et al., 2007), and the reliability of the client (Lin, 2008).

#### Social cognitive theory

Social cognition has been defined as “the domain of cognition that involves the perception, interpretation, and processing of social information” and “the space of discernment that includes the observation, understanding, and handling of social data” (Penn et al., 1997). Albert Bandura’s social learning hypothesis was formed into the SCT in 1986, which posits that learning happens in a social setting with dynamic and corresponding communication between the individual, conditions, and conduct. SCT’s unique feature is its accentuation of social impact and outer and inner social support (Bandura, 1977a). The theory considers the extraordinary manner by which people obtain and develop conduct, as well as the social condition wherein they play out this conduct. The hypothesis also considers an individual’s previous encounters, which factor in whether certain future social activity will occur. These previous encounters impact fortifications, desires, and hopes, all of which shape whether an individual will take part in a particular conduct and the reasons for the individual’s participation (Bandura, 1977a).

#### Expectation confirmation theory

**Fig. 2:**
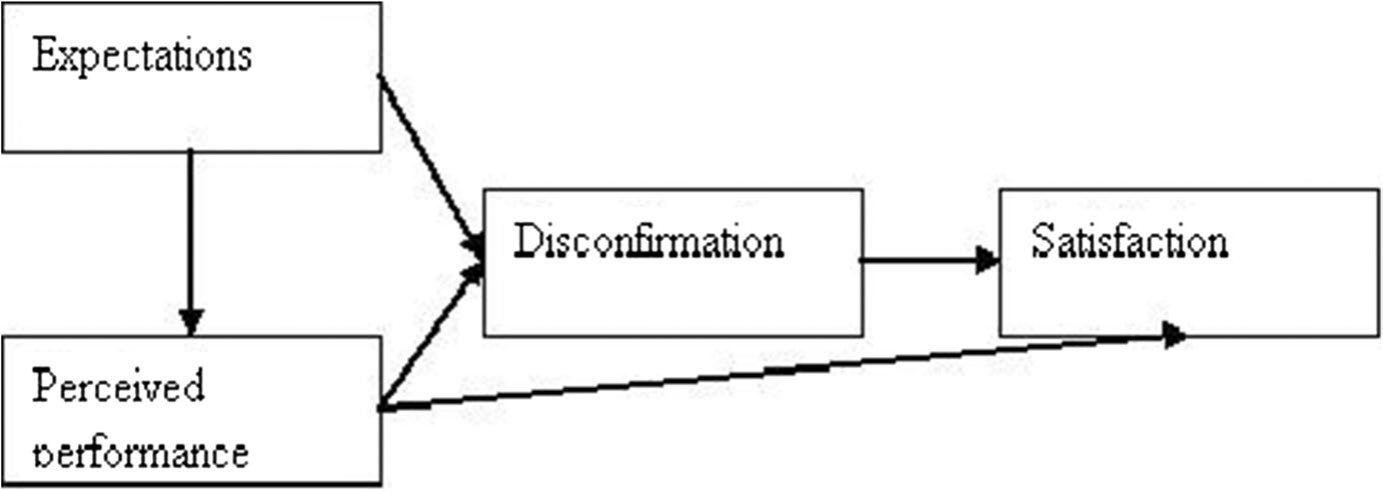
Expectation confirmation theory (Oliver, 1980)

ECT is an intellectual hypothesis that attempts to clarify post-buy or post-reception fulfillment as a component of desires, execution, and disconfirmation of convictions (Oliver, 1980), and, relatedly, the satisfaction of desires leads to positive changes in resolve. Figure 2 illustrates the basic model of ECT, including its four principles of expectations, perceived performance, disconfirmation, and fulfillment.

ECT has been used to determine factors affecting fulfillment with some studies highlighting specific settings (McKinney et al., 2002). Many have utilized subsequent fulfillment to decide whether to proceed with the utilization of a framework (Liao et al., 2007), and yet others have combined ECT with other theories to seek a more complete image of how fulfillment is determined (Sorebo & Eikebrokk, 2008).

## Research framework and hypothesis

**Fig. 3:**
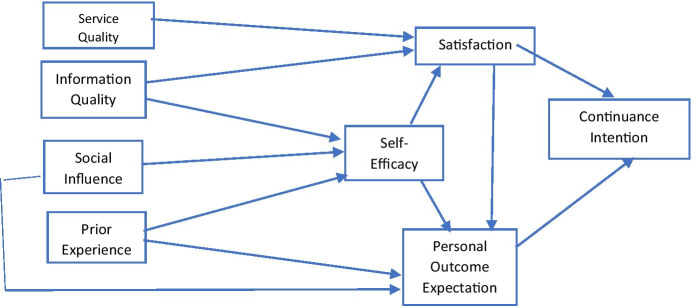
Proposed framework

This study developed a framework to investigate the critical factors influencing students’ satisfaction and their continuance intention to use LMSs (Fig. 3). The framework integrated the D&M model, SCT, and ECT, and comprised eight constructs and 12 hypotheses designed to examine the relationships between these constructs.

### Service quality

According to Parasuraman et al. (1988), service quality is “the consumer’s judgment about an entity’s overall excellence superiority.” Grönroos (1984) referred to service quality as “the outcome of an evaluation process, where the consumer compares his expectations with the service he perceives he has received.” Consequently, this study hypothesizes that:

- H1: Service quality positively influences students’ satisfaction

### Information quality

DeLone & McLean (2003) examined the connection between the nature of data and individual effects that can be estimated by precision, practicality, satisfaction, importance, and consistency. Moreover, according to Alsabawy et al. (2016), the nature of data can be estimated by utilizing the components of significance, accessibility, ease of use, understandability, and compactness. Information quality concerns whether it creates and conveys the framework (DeLone & McLean, 1992) and is related to the issues of online content. Past experiments have found that information quality is emphatically connected with client satisfaction. Hence, the nature of information affects users’ capacity to access and read this information, framing their result desire to return to a site and characterizing their general satisfaction. Therefore, the following hypotheses are proposed:

- H2: Information quality positively influences students’ satisfaction with LMSs.
- H3: Information quality positively influences self-efficacy.

### Social influence

Social impact represents how much others’ convictions impact a person in their choice to utilize a framework (Venkatesh et al., 2003). Various estimations have been used to survey the adequacy of social effects on people’s conduct. Social impact can be viewed as how much peers impact the utilization of a framework, either positively or negatively (Bandura, 1986; Chan et al., 2010). Abstract standard is another term that has been used to describe the relationship with innovation reception (Venkatesh et al., 2003). Furthermore, Bandura (1977b) noted that enactive dominance, vicarious experience, and verbal influence, including passionate excitement, could impact self-adequacy since individuals are components of society (Bandura, 1986). Therefore, the following hypotheses are proposed:

- H4: Social influence positively influences self-efficacy.
- H5: Social influence positively influences personal outcome expectations.

### Prior experience

Bandura alluded to related knowledge as enactive dominance (Bandura, 1986; Johnson & Marakas, 2000). Related knowledge is one’s mental impression of the last involvement with an online system (Hussein et al., 2011), and fruitful past encounters can build one’s trust in its self-viability and usefulness. Besides social impact, related knowledge is key to self-adequacy (Compeau & Higgins, 1995). In SCT, Bandura (1986) partnered earlier execution, self-adequacy, and result desires. Chan et al. (2010) used similarity—which demonstrates related knowledge or inclinations—as a construct that impacts execution hopes across important online systems. Bhattacherjee & Premkumar (2004), meanwhile, found that exhibition disconfirmation is a significant determinant of client satisfaction within internet banking frameworks. In another investigation, Hsu et al. (2004) found a positive connection between clients’ earlier disconfirmations, their satisfaction levels with previous Internet use, and their result desires for proceeding with use. Thus, the congruity of use can be utilized as a measure to assess client satisfaction dependent on experience. Accordingly, the study proposes the following hypotheses:

- H6: Prior experience positively influences self-efficacy.
- H7: Prior experience positively influences personal outcome expectation.

### Satisfaction

Satisfaction refers to the emotional assessment of the different outcomes that can also be applicable to viewpoints seen as lovely or upsetting. Satisfaction considers shared sentiments based on past encounters with online systems (Oliver, 1980) and has been viewed as a suitable variable in examinations of online help (Chan et al., 2010) and the success of online administration. As per ECT, satisfaction with assistance that is offered is a solid indicator of clients’ duration goals and may impact framework appropriation (Bhattacherjee & Premkumar, 2004). Thus, this study proposes the following hypotheses:

- H8: Satisfaction positively influences personal outcome expectations.
- H9: Satisfaction positively influences continuous intention to use LMSs.

### Self-efficacy

The concept of self-efficacy focuses mainly on individual perceptions of efficacy and is an important factor influencing what individuals can achieve (Bandura, 1977b). The performance of dissimilar people with a similar set of skills, as well as the performance of the same person in different situations, depends on changes in their perceptions of themselves. Compeau & Higgins (1995, 191) characterized self-viability in the ICT setting as “a person’s view of their capacity to utilize PCs in the achievement of an errand.” Assignment-explicit PC self-efficacy alludes to “a person’s impression of viability in performing explicit PC-related undertakings inside the space of general processing” (Marakas et al., 1998, 128). Self-viability is a kind of self-evaluation that impacts choices about certain practices and represents a proportion of the exertion put into something during troublesome occasions. Self-adequacy or convictions about one’s capacity to perform a specific task or conduct relates to satisfaction (SAT), continuation aim (CI), individual result desire (POE), data quality (intelligence level), system quality (SQ), social impact (SI), related knowledge (PE), and self-viability (SE) (Alruwaie et al., 2020; Bandura, 1986; Compeau & Higgins, 1995). SCT contends that self-viability recognition impacts a person’s resultant desires (Compeau & Higgins, 1995). As per Bandura’s (1986) social intellectual hypothesis, people form their view of self-adequacy toward an assignment in light of data they obtain from past understandings (commonality with comparative exercises), vicarious understanding (through others), social help and support, their mental states and demeanors toward a task, and decisions on signs they perceive from a similar source. As such, people decipher and gauge outcomes dependent on their self-viability convictions. Therefore, the following hypotheses are proposed:

- H10: Self-efficacy positively influences students’ satisfaction.
- H11: Self-efficacy positively influences personal outcome expectations.

### Personal outcome expectations

Individual result desire is “an individual’s gauge that a given conduct will prompt a specific result” (Bandura, 1977b, 193), while result hopes are the outcomes of a specific activity (Bandura, 1986). There is a connection between the necessary aptitudes, for example, physical capacity (self-adequacy) and mental capacity (individual result desires); physical capacity normally emerges before mental capacity (Bandura, 1977b, 1986). “People expecting constructive advantages from utilizing PCs will be more exceptionally energetic than those not anticipating any advantages, while likewise being relentless in their endeavors to find out additional” (Compeau & Higgins, 1995, 122); thus, positive outcomes derive from result viability and result desires as individuals regularly settle on activity-based choices that are established within their abilities (Bandura, 1986). The intensity of constructive results can spur individuals to attempt tasks and search for complex assignments, and as they are fulfilled, feel secure about managing possible future events (Johnson & Marakas, 2000). Conduct is controlled by result desires if there are uncontrolled viability convictions. To address this important issue, this study proposed the following hypothesis:

- H12: Personal outcome expectations positively influence students’ continuous intention to use LMSs.

## Research methodology

### Measures

Surveys are the most popular quantitative research strategy for data collection and are typical in social science research. The present research applied a five-point Likert scale questionnaire, which is a quantitative data collection tool (Jamieson, 2004). Each item is a statement to which the respondent must express a degree of agreement or disagreement between 1 and 5 (e.g., 1 = strongly agree, 2 = agree, 3 = neither agree nor disagree, 4 = disagree, 5 = strongly disagree). Four independent constructs were examined—service quality, information quality, social influence, and prior experience—as well as four dependent constructs: satisfaction, self-efficacy, personal outcome expectation, and continuous intention. Each of the constructs was measured through multiple items.

### Pre-test and pilot test

Pre-tests allow problems that cannot be predicted during the application of the questionnaire to be taken into account, helping the researcher obtain better results. Pilot testing, meanwhile, aims to establish if the research instrument will work as a live project through its application with a small pilot population and can reduce weaknesses in the questions before a field launch. Initially, 50 questionnaires were sent to the respondents, and the exploratory factor examination results indicated that each of the eight elements had good dependability and legitimacy. A few concerns raised during the pilot study, related to the clarity of guidelines and questions, the general design, and other minor remarks, were addressed. All ambiguities were removed to guarantee the meaningfulness of the scales.

### Sample

Sampling is the process of selecting a set of individuals from a population so as to be able to represent the whole population in a study (McDonald et al., 2015). Probability sampling is the most frequently used method for drawing robust and reliable conclusions. A larger sample size better represents the population (Collis & Hussey, 2013), and larger data sets improve the quality of the research outcomes by enhancing generalizability and reliability. We sent our questionnaire to 400 students and received 181 completed responses. The sample was chosen randomly from among UK-based university students in the first year of an undergraduate program.

### Data collection

The questionnaire was distributed online via Google Forms, emails, and Moodle, and respondents’ personal information was subsequently deleted to ensure confidentiality. Table 2 presents the questionnaire constructs and related items.

**Table 2:** Constructs and their respective items

Constructs	Questions
Information quality	Through Blackboard, I get the information I need in time
	Information provided by Blackboard meets my needs
	Information provided by Blackboard is in a useful format
	Information provided by Blackboard is clear
	Information provided by Blackboard is accurate and up to date
Service quality	Blackboard makes it easy to find what I need
	Blackboard is simple to use
	Blackboard is always available (24/7)
	Blackboard launches and runs right away
	Blackboard has technical support representatives available online
Social Influence	People who influence my behavior would think that I should the Blackboard to learn during the COVID-19 pandemic
	People who are important to me would think that I should use Blackboard
	People who are in my social circle would think that I should use Blackboard
Prior Experience	The information quality on Blackboard was better than I expected
	The service quality of Blackboard was better than I expected
	Overall, the quality of Blackboard was better than I expected
Satisfaction	I am satisfied with the use of Blackboard
	I am satisfied with the service quality of Blackboard
	Overall, I am satisfied with the quality of the Blackboard system
Self-efficacy	I feel confident finding my way around Blackboard
	I feel confident looking for information by querying Blackboard
	I feel confident e-mailing the Blackboard system
	I find it easy to use Blackboard
	Overall, I am confident in my ability to access the Blackboard system
Personal Outcome Expectations	If I use Blackboard, I can gather more complete and timely information when compared with the traditional education system
	If I use Blackboard, I will increase my sense of education
	If I use a computer to access Blackboard, I will be better organized, compared to using traditional education systems
	If I use Blackboard, I will spend less time, compared to traditional education systems
Continuance Intention	I intend to continue using Blackboard in the future
	I will continue using Blackboard in the future
	I will regularly use Blackboard in the future

## Data analysis and findings

The proposed model was assessed and validated using structural equation modeling (SEM), in particular, the partial least square method since the dataset did not follow a multivariate normal distribution and the sample size was small.

### Measurement model evaluation

This study’s research model was constructed with 31 indicator items and comprised eight dimensions of student satisfaction and continuous use characteristics. Cronbach’s reliability, composite reliability, and convergent validity tests were used to evaluate the measurement model (Hair et al., 2017).

All latent variables had Cronbach’s reliability values that were much larger than the minimum acceptable level of 0.4 and close to the preferred level of 0.7 (Table 3). The composite reliability was also larger than 0.7; thus, high levels of internal consistency reliability were demonstrated among all eight reflective latent variables. Moreover, each latent variable’s average variance extracted (AVE) was evaluated to check convergent validity. All the AVE values in the measurement model were greater than the acceptable threshold of 0.5; therefore, convergent validity was confirmed (Table 3). Table 4 presents the discriminant validity for all eight constructs as the purpose of discriminant validity is to test whether the latent variables differ from each other by comparing the inter-construct correlations with the square roots of their respective average variances extracted. When comparing the square roots of the AVEs with the other values on each column, the square roots of the AVEs for each latent variable must be greater than any correlation relating to each latent variable (Hair et al., 2017).

**Table 3:** Cronbach’s Alpha, composite reliability, and AVE

	Cronbach’s Alpha	Composite Reliability	AVE
CI	0.927	0.954	0.873
IQ	0.954	0.965	0.845
PE	0.935	0.958	0.885
POE	0.940	0.957	0.849
SAT	0.920	0.949	0.862
SE	0.948	0.960	0.828
SI	0.943	0.964	0.898
SQ	0.954	0.965	0.847

**Table 4:** Discriminant validity

	CI	IQ	PE	POE	SAT	SE	SI	SQ
CI	0.934							
IQ	0.914	0.919						
PE	0.902	0.830	0.941					
POE	0.885	0.900	0.902	0.921				
SAT	0.925	0.845	0.918	0.866	0.928			
SE	0.912	0.911	0.898	0.887	0.915	0.910		
SI	0.898	0.889	0.912	0.921	0.881	0.909	0.948	
SQ	0.911	0.834	0.923	0.906	0.915	0.906	0.899	0.920

According to Hair et al. (2017), the outer weight explains how much each indicator loads on the respective latent variables. As shown in Table 5, all indicators for the dimensions in each construct resulted in a loading factor (λ) greater than 0.5; therefore, no indicator was excluded from the model.

**Table 5:** Outer weights

	Outer Weights	P-Values
CI1 <—CI	0.924	0.000
CI2 <—CI	0.949	0.000
CI3 <—CI	0.930	0.000
IQ1 <—IQ	0.898	0.000
IQ2 <—IQ	0.939	0.000
IQ3 <—IQ	0.939	0.000
IQ4 <—IQ	0.907	0.000
IQ5 <—IQ	0.911	0.000
PE1 <—PE	0.905	0.000
PE2 <—PE	0.959	0.000
PE3 <—PE	0.957	0.000
POE1 <—POE	0.891	0.000
POE2 <—POE	0.946	0.000
POE3 <—POE	0.952	0.000
POE4 <—POE	0.895	0.000
SAT1 <—SAT	0.916	0.000
SAT2 <—SAT	0.947	0.000
SAT3 <—SAT	0.921	0.000
SE1 <—SE	0.892	0.000
SE2 <—SE	0.923	0.000
SE3 <—SE	0.900	0.000
SE4 <—SE	0.915	0.000
SE5 <—SE	0.921	0.000
SI1 <—SI	0.926	0.000
SI2 <—SI	0.972	0.000
SI3 <—SI	0.945	0.000
SQ1 <—SQ	0.868	0.000
SQ2 <—SQ	0.963	0.000
SQ3 <—SQ	0.941	0.000
SQ4 <—SQ	0.902	0.000
SQ5 <—SQ	0.924	0.000

### Structural model evaluation

The structural model was evaluated and examined by considering both the coefficient of determination and path coefficient values to assess the statistical significance of each hypothesis (Hair et al., 2017). The R-Square value was 0.884 for the Continues Intention (CI) endogenous latent variable (Table 6), meaning that the four latent variables, Personal Outcome Expectation (POE), Continues Intention (CI), Self-Efficacy (SE), and Satisfaction (SAT), explained 88.4% of the variance of the Continues Intention (CI) latent variable. The R-Square Adjusted value explained 88.2% of the Continues Intention (CI) latent variable.

**Table 6:** Coefficients of determination

	R-Square	R-Square Adjusted
CI	0.884	0.882
POE	0.875	0.872
SAT	0.912	0.910
SE	0.878	0.876

**Fig. 4:**
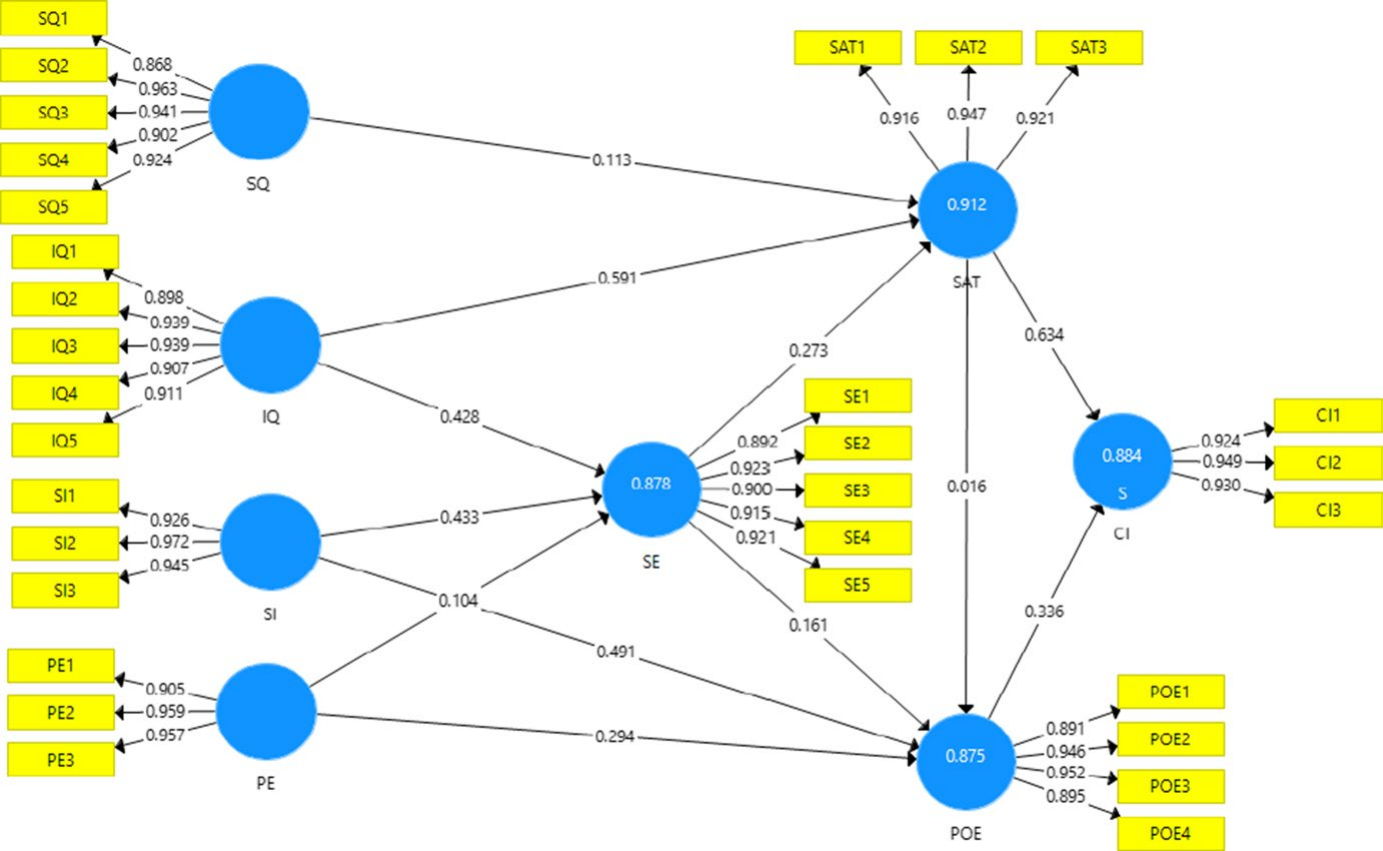
Structural model with path coefficient values

Table 7 presents the results of the path coefficients and *p*-values for all the proposed hypotheses. The path coefficients provide the significance of the hypothesized relations connecting the constructs. Four hypotheses were not supported since the *p*-values were greater than 0.05, while all other hypotheses were supported with *p*-values of less than 0.05. While service quality was proposed to have a significant influence on students’ satisfaction, the results highlighted its insignificant influence during the COVID-19 pandemic. Figure 4 presents the final measurements and structural model.

**Table 7:** Hypotheses’ path coefficients and *p*-values

	Path Coefficients	*P*-Values
IQ—> SAT	0.591	0.000
IQ—> SE	0.428	0.000
PE—> POE	0.294	0.003
PE—> SE	0.104	0.367
POE—> CI	0.336	0.000
SAT—> CI	0.634	0.000
SAT—> POE	0.016	0.819
SE—> POE	0.161	0.167
SE—> SAT	0.273	0.001
SI—> POE	0.491	0.000
SI—> SE	0.433	0.000
SQ—> SAT	0.113	0.348

## Discussion

This study analyzed the factors affecting students’ satisfaction and their continuance intention toward LMS use in higher education during the COVID-19 pandemic. A proposed model was developed based on the integration of SCT, ECT, and the D&M model. In total, eight constructs were selected for the model: service quality, information quality, social influence, experience, satisfaction, self-efficacy, personal outcome expectations, and continuance intention to use LMS. The proposed model was tested in the field of e-learning, based on the study of e-governments by Alruwaie et al. (2020). The relationships between the constructs were analyzed using SEM.

### DeLone and McLean’s IS success model

Four factors from the D&M model were used: service quality, information quality, satisfaction, and continuous intention of use. Service quality did not significantly influence UK higher education students’ satisfaction during the COVID-19 pandemic, a result that differs from extant studies as service quality in UK is high for all students compared to other countries. For example, Ohliati & Abbas (2019) highlighted a strong and significant influence of service quality on students’ satisfaction, while Alruwaie et al. (2020) revealed that students’ satisfaction with LMSs increased with effective service quality.

Information quality had a significant impact on students’ satisfaction in the present study, a finding that is similar to those in the literature (Alruwaie et al., 2020; Ohliati & Abbas, 2019. Thus, our findings reveal that the COVID-19 pandemic did not alter the significant importance of information quality on UK students’ satisfaction with LMSs.

The study also highlighted the significant impact of students’ satisfaction on their continuous intention to use LMSs. However, the results revealed the insignificant influence of satisfaction on personal outcome expectations during the pandemic, which again differed from other recent studies (Alruwaie et al., 2020). Furthermore, the study found that personal outcome expectations had a significant impact on students’ intention to use LMSs during the pandemic.

### Social cognitive theory

Two SCT factors were adopted in the present research model: self-efficacy and social influence. The results highlighted the significant influence of self-efficacy on students’ satisfaction, although it did not impact personal outcome expectations. A significant impact of social influence on both self-efficacy and personal outcome expectations was also found, further confirming most findings in the literature regarding the importance of social influence in users’ behavior (e.g., Alruwaie et al., 2020).

### Expectation confirmation theory

Finally, the results of this study reveal that personal outcome expectations have a significant influence on students’ continuous intention to use LMSs. Two factors influencing personal outcome expectations were also highlighted: social influence and prior experience. This study also emphasized the insignificant impact of self-efficacy and UK students' satisfaction on students’ outcome expectations, demonstrating that the COVID-19 pandemic influence UK students in this factor.

## Conclusion and research limitations

This study analyzed and examined the critical factors affecting students’ continuous use of LMSs in higher education during the COVID-19 pandemic. A structural research model was proposed and validated through an online survey. The results highlight that during the pandemic, service quality did not influence students’ satisfaction, while information quality and self-efficacy both had a significant influence. In addition, the findings highlighted that neither self-efficacy nor satisfaction impacted personal outcome expectations, although prior experience and social influence did demonstrating that the COVID-19 pandemic significantly influence UK students in this factor. The findings have significant practical implications for education developers, policymakers, and practitioners seeking to develop effective strategies for and improve the use of LMSs during COVID-19.

However, the study has three limitations. First, the data were collected from a small sample of UK students at one university; thus, the range of universities and the number of students using this system should be extended to improve the generalizability of the results. In addition, the study used quantitative research methodology, and using qualitative examination could reveal more explanations for the relationships between the proposed constructs. Therefore, further studies should support their quantitative findings with a qualitative approach. Finally, further studies with cross-sectional and cross-cultural approaches are required to increase the predictive value of LMSs.
